# Predicting Anthracycline-Induced Cardiotoxicity Using the HFA-ICOS Risk Score: A Validation Study From AIC Registry

**DOI:** 10.1016/j.jacasi.2026.04.036

**Published:** 2026-06-19

**Authors:** Atsuko Suzuki, Keiko Inoue, Tomoko Machino-Ohtsuka, Yoko Nakazawa, Noriko Iida, Rumi Sasamura, Hiroko Bando, Nobutaka Tasaka, Junko Ohtsuka, Yuma Shibutani, Yuki Ishizuka, Takuro Imaoka, Tomoko Ishizu, Ikuo Sekine, Kazuko Tajiri

**Affiliations:** a Department of Clinical Laboratory, National Cancer Center Hospital East, Kashiwa, Japan; b Department of Cardiology, Institute of Medicine, University of Tsukuba, Tsukuba, Japan; c Department of Cardiology, Mito Kyodo General Hospital, Mito, Japan; d Kenpoku Medical Center, Takahagi Kyodo Hospital, Takahagi, Japan; e Department of Medical Technology, Faculty of Health Sciences, Tsukuba International University, Tsuchiura, Japan; f Clinical Laboratory, University of Tsukuba Hospital, Tsukuba, Japan; g Department of Breast and Endocrine Surgery, Institute of Medicine, University of Tsukuba, Tsukuba, Japan; h Department of Obstetrics and Gynecology, Hitachi General Hospital, Hitachi, Japan; i Department of Cardiology, National Cancer Center Hospital East, Kashiwa, Japan; j Department of Cardiology, University of Tsukuba Hospital, Tsukuba, Japan; k Department of Pharmacy, National Cancer Center Hospital East, Kashiwa, Japan; l Department of Cardiology, Tokyo Metropolitan Bokutoh Hospital, Tokyo, Japan; m Department of Medical Oncology, Institute of Medicine, University of Tsukuba, Tsukuba, Japan; n Tsukuba Life Science Innovation Program, School of Integrative and Global Majors, University of Tsukuba, Tsukuba, Japan

**Keywords:** cardiomyopathy, cardio-oncology, CTRCD, echocardiography, onco-cardiology, heart failure

Anthracyclines are widely used chemotherapeutic agents, but their effectiveness can be limited by cancer therapy–related cardiac dysfunction (CTRCD). Recently, a predictive risk score to estimate baseline cardiovascular risk in patients starting cardiotoxic therapies, including anthracyclines, was developed by the Heart Failure Association (HFA) and the International Cardio-Oncology Society (ICOS)^1^ and is recommended in the 2022 European Society of Cardiology guidelines on cardio-oncology.^2^ The score incorporates clinical factors such as patient-related cardiovascular risk factors, prior cardiovascular disease, and cancer therapy–related variables to estimate baseline cardiotoxicity risk.^2^ However, this model has limited validation, particularly in Asian populations. Therefore, we performed an external validation of the HFA-ICOS risk score using the AIC Registry in Japan.^3^^,^^4^

This study followed the Transparent Reporting of a Multivariable Prediction Model for Individual Prognosis or Diagnosis statement for prediction model validation.^5^ The study was based on data from patients with cancer who were receiving anthracycline chemotherapy and were prospectively enrolled in the AIC Registry between July 2016 and October 2021.^3^^,^^4^ Patients with baseline left ventricular ejection fractions (LVEF) of <50%, moderate or greater valvular disease, congenital heart disease, or cardiomyopathy were excluded. Clinical and echocardiographic data were prospectively collected before starting anthracyclines and during follow-up. The study was conducted in accordance with the Declaration of Helsinki and approved by the Institutional Review Board of the University of Tsukuba Hospital (H28-06). Written informed consent was obtained from all patients.

The primary endpoint was CTRCD during the 2-year follow-up period, defined as a ≥10% reduction in LVEF to <55%.^6^ The follow-up period was based on the AIC Registry design,^4^ a time frame considered clinically relevant for cardiotoxicity. The HFA-ICOS score was calculated using baseline variables, and patients were stratified into low-, moderate-, and high-risk groups according to predefined thresholds.^1^ There were no missing data required to calculate the score.

Competing risk analyses were performed treating death as a competing event. Cumulative incidence was compared using Gray’s test, and Fine-Gray models were used to estimate subdistribution HRs. Discrimination was assessed using Uno’s C statistic, with the area under the receiver-operating characteristic curve (AUC) as a supplementary measure. Sensitivity, specificity, positive predictive value, and negative predictive value were evaluated at 2 years. Calibration was assessed by comparing predicted and observed risks across risk groups. Clinical utility was evaluated using decision curve analysis to estimate net benefit across a range of threshold probabilities. Statistical significance was set at a 2-sided *P* value <0.05. Analyses were performed using R version 4.4.2 (R Foundation for Statistical Computing) with RStudio (Posit) or EZR (https://www.jichi.ac.jp/usr/hema/EZR/statmedEN.html).^7^

A total of 339 patients were included in this study. The median age was 55 years (Q1-Q3: 46-66 years), and 93% (315 of 339) were women. Most had breast cancer (290 of 339 [86%]) or hematologic malignancies (40 of 339 [12%]), and anthracycline regimens were administered according to standard clinical protocols. Detailed information on cancer types, cumulative anthracycline doses, and treatment duration has been previously reported.^4^ According to the HFA-ICOS score, 166 of 339 patients (49.0%) were classified as low risk, 140 of 339 (41.3%) as moderate risk, and 33 of 339 (9.7%) as high risk.

Within 2 years, 27 of 339 patients (8.0%) developed CTRCD. Cumulative incidence differed across risk groups (*P* = 0.044, Gray’s test); however, the low- and moderate-risk groups exhibited similar trends (Figure 1A). Fine-Gray analysis revealed that high-risk patients had a greater risk for CTRCD than low-risk patients (subdistribution HR: 3.50;95% CI: 1.31-9.35; *P* = 0.012) (Figure 1B). In contrast, patients at moderate risk did not show a statistically significant increase in risk compared with low-risk patients.

**Figure 1:**
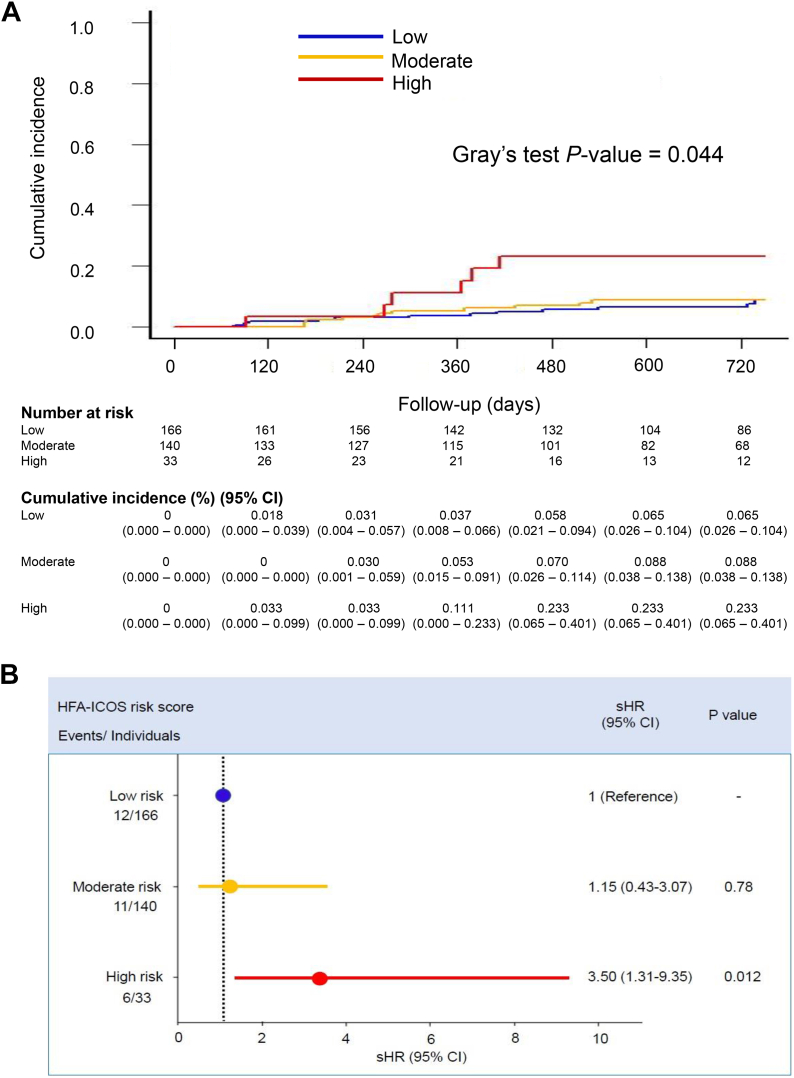
CTRCD Risk Across HFA-ICOS Groups (A) Cumulative incidence curves for cancer therapy–related cardiac dysfunction (CTRCD) according to Heart Failure Association (HFA)–International Cardio-Oncology Society (ICOS) risk groups, treating death as a competing event. (B) Fine-Gray subdistribution hazard analysis of CTRCD according to HFA-ICOS risk groups. sHR = subdistribution HR.

At 2 years, HFA-ICOS risk stratification demonstrated sensitivity of 19.4% (95% CI: 7.5%-37.5%), specificity of 91.2% (95% CI: 87.5%-94.1%), positive predictive value of 18.2% (95% CI: 7.0%-35.5%), and negative predictive value of 91.8% (95% CI: 88.2%-94.6%) for predicting CTRCD when comparing high-risk patients with those classified as low or moderate risk.

The discriminative ability of the HFA-ICOS score was modest. Uno’s C statistic for predicting 2-year CTRCD was 0.594 (95% CI: 0.490-0.699). For reference, the AUC for predicting 2-year CTRCD was 0.599 (95% CI: 0.529-0.727). Predicted and observed risks were generally well aligned across risk categories, although the low- and moderate-risk groups showed similar estimates. Decision curve analysis showed limited net clinical benefit, comparable with a treat-none strategy.

To date, 2 validation studies of the HFA-ICOS score have been reported.^8^^,^^9^ Chen et al^9^ prospectively enrolled breast cancer patients receiving anthracycline therapy and assessed the predictive accuracy for CTRCD, defined as a ≥10% reduction in LVEF to >53%. The incidence of CTRCD was 10.1% to 12.8% in the low-risk group, 40.0% to 48.0% in the moderate-risk group, and 53.3% to 55.6% in the high-risk group. The model showed modest discrimination (C indexes of 0.633-0.642), consistent with our findings. Interestingly, in their cohort, CTRCD-free survival did not differ between high- and moderate-risk subjects, in contrast to our findings. In another study from the CARDIOTOX (Cardiovascular Toxicity Induced by Antitumoral Drugs: Risk Assessment and Early Diagnosis) registry,^8^ the incidence of symptomatic or severe or moderate asymptomatic CTRCD was 2.3%, 6.7%, 19.7%, and 40% in the low-risk, moderate-risk, high-risk, and very high risk groups, respectively. Discrimination was higher (AUC: 0.78; Uno’s C statistic = 0.78) than in our cohort. These findings suggest that although the HFA-ICOS score can stratify CTRCD risk, its performance varies across populations and definitions of CTRCD. Nonetheless, patients classified as high risk or above consistently show a high incidence of CTRCD and should be monitored carefully.

This study had several limitations. First, although prospective in nature, the HFA-ICOS score was applied retrospectively. Second, as the AIC Registry excluded patients with histories of cardiovascular disease, the cohort included a relatively small number of high-risk patients, and none of the patients were classified as very high risk. Third, the definition of CTRCD used in this study differs from those in the current European Society of Cardiology guidelines.^2^

In conclusion, the HFA-ICOS score effectively identified patients at high risk for CTRCD among those receiving anthracycline chemotherapy in the AIC Registry. However, it has limited predictive ability to discriminate between mild- and moderate-risk groups.

### Data availability

The data underlying this study will be shared on reasonable request from the corresponding author.

## Funding Support and Author Disclosures

This work was supported by grants from the Japan Society for the Promotion of Science KAKENHI (23K07518 and 24K02250), the Agency for Medical Research and Development (24ek0109755h0001, 25ek0109795s0701, and 25ak0101282h0301), and the National Cancer Center Research and Development Fund (2023-A-12, 2025-S-02, and 2026-A-08). The authors have reported that they have no relationships relevant to the contents of this paper to disclose.
